# Molecular Subtyping for Predicting Pathological Upstaging and Survival Outcomes in Clinically Organ-confined Bladder Cancer Patients Undergoing Radical Cystectomy

**DOI:** 10.1016/j.euros.2024.12.009

**Published:** 2025-01-27

**Authors:** Joep J. de Jong, James A. Proudfoot, Siamak Daneshmand, Robert S. Svatek, Vikram Narayan, Elai Davicioni, Shreyas Joshi, Aaron Dahmen, Roger Li, Brant A. Inman, Paras Shah, Iftach Chaplin, Jonathan Wright, Ewan A. Gibb, Yair Lotan

**Affiliations:** a Erasmus University Medical Center, Rotterdam, The Netherlands; b Veracyte Inc., San Diego, CA, USA; c Department of Urology, USC/Norris Comprehensive Cancer Center, University of Southern California, Los Angeles, CA, USA; d University of Texas Health San Antonio, San Antonio, TX, USA; e Emory University School of Medicine, Atlanta, GA, USA; f Moffitt Cancer Center, Tampa, FL, USA; g Western University, London, ON, Canada; h Mayo Clinic, Rochester, MN, USA; i University of Texas Southwestern, Dallas, TX, USA; j University of Washington, Seattle, WA, USA

**Keywords:** Molecular subtyping, Clinical staging, Bladder cancer, Radical cystectomy

## Abstract

**Background and objective:**

Many patients with bladder cancer are understaged. Previous work revealed that molecular subtyping using Decipher Bladder improves clinical staging. This multicenter validation study evaluated Decipher Bladder for upstaging in patients who underwent radical cystectomy (RC) without neoadjuvant therapy.

**Methods:**

The Decipher Bladder genomic subtyping classifier (GSC; Veracyte, San Diego, CA, USA) was performed on bladder tumor specimens from patients with high-grade, clinically organ-confined (cTa-T2N0M0) urothelial carcinoma who subsequently underwent RC without neoadjuvant chemotherapy. The primary endpoint was pathological upstaging to non–organ-confined (NOC) disease (pT3+ and/or N+) at RC. The secondary endpoints included overall survival (OS) and pathological upstaging to MIBC+ disease (pT2+ and/or N+) at RC within clinically non–muscle-invasive bladder cancer (cNMIBC) cases.

**Key findings and limitations:**

A total of 226 patients (134 cNMIBC [cTa/Tis/T1] and 92 cT2) were analyzed from eight participating institutions. Upstaging to NOC disease was observed in 33% of patients (19% for cNMIBC and 53% for cT2). Molecular subtyping identified 138 luminal and 88 nonluminal tumors. Rates of upstaging to NOC were 41% in nonluminal and 28% in luminal tumors (univariable *p* = 0.04), which was not independently significant after adjusting for clinical variables. Upstaging to MIBC+ in cNMIBC patients was lower in luminal versus nonluminal tumors (32% vs 51%, multivariable *p* = 0.03). Patients with nonluminal tumors had worse OS on multivariable analyses (*p* < 0.05). Limitations include retrospective design and sample size.

**Conclusions and clinical implications:**

Luminal tumors represent less aggressive disease, reflected by lower rates of pathological upstaging and favorable OS with RC compared with nonluminal tumors.

**Patient summary:**

Molecular subtyping suggests that in clinically non–muscle-invasive bladder cancer, luminal tumors harbor less aggressive disease, as reflected by lower rates of pathological upstaging to muscle-invasive disease and favorable outcomes with radical cystectomy, in comparison with nonluminal bladder cancer.

## Take Home Message

In patients with clinically organ-confined bladder cancer, luminal tumors show lower rates of pathological upstaging at radical cystectomy. Furthermore, patients with luminal bladder cancer have better overall survival after radical cystectomy without neoadjuvant therapy than patients harboring nonluminal bladder cancer.

## 1 Introduction

Radical cystectomy (RC) surgery with or without neoadjuvant chemotherapy (NAC) is one of the recommended treatment options for muscle-invasive bladder cancer (MIBC; cT2-T4) but is also recommended for patients with high-risk non–muscle-invasive bladder cancer (NMIBC) [1]. NAC is recommended for clinical MIBC, but underutilized due to concerns for toxicity, perceived modest survival benefit, and a risk of overtreatment in true organ-confined disease (pT2N0M0) [2]. Accurate clinical staging of bladder cancer remains a significant challenge, as pathological upstaging at RC is common and increases the risk of mortality significantly [3]. Importantly, in patients undergoing RC without neoadjuvant therapy, pathological upstaging at RC represents a missed opportunity to administer systemic therapy prior to surgery. There is some evidence that NAC may be more beneficial in patients with non–organ-confined (NOC) disease [4]. Hence, tools to improve staging accuracy for patients with bladder cancer are greatly needed to improve patient management and care.

Molecular subtyping can be used to divide bladder cancer into several classes, each with a unique biological and clinical presentation. Subtyping models generally split bladder cancer into basal and luminal subtypes, with different models providing additional second-order splitting for more precise classification [5]. Previous work has shown that patients with nonluminal subtype have worse prognosis but are more responsive to cisplatin-based chemotherapy, while the luminal subtype tends to have more favorable survival outcomes but are less responsive to cisplatin [6], [7]. Additionally, a multicenter trial evaluating patients with cT1/2 bladder cancer who underwent cystectomy without NAC found that luminal tumors have lower rates of pathological upstaging to NOC disease at RC [8]. Taken together, these observations indicate that luminal tumors have a less aggressive disease presentation and may represent a tumor type where immediate RC without NAC is the treatment of choice. Contrarily, patients with nonluminal tumors may be at a higher risk of upstaging and derive a greater benefit from neoadjuvant therapies.

In this study, we assembled a multicenter cohort of patients to validate previously reported observations that luminal bladder tumors have lower rates of pathological upstaging at RC and that luminal bladder cancer is associated with better survival outcomes.

## 2 Patients and methods

### 2.1 Patient population

We retrospectively analyzed patients from eight selected US tertiary care cancer centers, who were diagnosed with high-grade cTa-2N0M0 bladder cancer and were treated with RC and bilateral pelvic lymphadenectomy within 4 mo of diagnosis. Eligible patients had formalin-fixed and paraffin-embedded (FFPE) cancer specimens from transurethral resection of the bladder tumor (TURBT) available for genomic analyses, did not receive neoadjuvant systemic therapy, had no presence of variant histology (eg, micropapillary) other than nonpredominant mixed urothelial with squamous or glandular differentiation, and did not have presence of cancer in a bladder diverticulum. All patients underwent TURBT, bimanual examination, and cross-sectional imaging prior to cystectomy on which they had no signs of extravesical disease and/or hydronephrosis. Institutional review board approval was obtained from each of the participating institutions before conducting this retrospective study.

### 2.2 Specimen collection and processing

Specimen collection and sample processing were conducted as described previously [8]. The Decipher Bladder assay, a clinical-grade whole-transcriptome assay for FFPE specimens (Veracyte, San Diego, CA, USA), was used to generate GSC subtypes (luminal, basal, claudin low, infiltrated luminal, and neuroendocrine like), based on previously validated signatures [7], [9]. In all patients, TURBT prior to cystectomy was used for molecular analyses.

### 2.3 Gene expression analyses

Heatmaps were used to visualize differences between subgroups, using bladder cancer biomarker genes. R package consensus MIBC and BLCAsubtyping were applied to classify tumors among The Cancer Genome Atlas (TCGA) 2017 and consensus molecular subtypes [10].

### 2.4 Statistical analyses

The prespecified primary endpoint of this validation study was pathological upstaging to NOC disease at RC, defined as the presence of pT3–4 or pN1–3 bladder cancer. Secondary endpoints included upstaging to MIBC (pT2–4 or pN+) within the clinically non–muscle-invasive bladder cancer (cNMIBC; Ta, T1, or Tis) subgroup, and overall survival (OS) following surgery. Descriptive statistics with medians and interquartile ranges (IQRs), or frequencies and proportions were presented, as appropriate. Univariable and multivariable logistic mixed models were used to evaluate the association between genomic subtype and pathological upstaging, with covariate adjustment for age, sex, and clinical T stage and an institution-level random intercept, with odds ratios (ORs) and their associated 95% confidence intervals (CIs) reported. Univariable and multivariable Cox proportional hazard models were fit for OS with similar covariate adjustment, with hazard ratios (HRs) and their associated 95% CIs reported. The Kaplan-Meier method was used to visualize differences in survival outcomes between subgroups. All statistical tests were two sided, and the reported significance level was 0.05. Analyses were performed in R v4.3.3.

## 3 Results

### 3.1 Study cohort

A total of 236 patients were identified, of whom seven failed genomic quality control and three were clinically node positive, resulting in a final cohort size of 226 patients, of whom 42 (19%) were female. The median time interval between TURBT and RC was 1.4 mo (IQR 1.0–2.0 mo), and all patients were treated with RC between 2003 and 2020. The study population comprised 134 (59%) cNMIBC and 92 cT2 cases, and the median age at RC was 4 yr higher for cT2 patients than for cNMIBC patients (Supplementary Table 1). Pathological upstaging to NOC disease was observed in 25 (19%) and 49 (53%) patients with cNMIBC and cT2 disease, respectively. Thirty-eight (17%) patients had positive lymph nodes at RC, including 12 (9%) and 26 (28%) patients with cNMIBC and cT2, respectively. Thirty-six deaths were observed in cNMIBC patients and 34 were observed in cT2 patients. The median follow-up time for censored patients in the cohort was 33 mo (IQR: 18–41 mo). Of note, in five patients, lymphovascular invasion was reported as present at TURBT, of whom four had pathological upstaging to NOC disease at RC.

### 3.2 Molecular subtyping

**Fig. 1:**
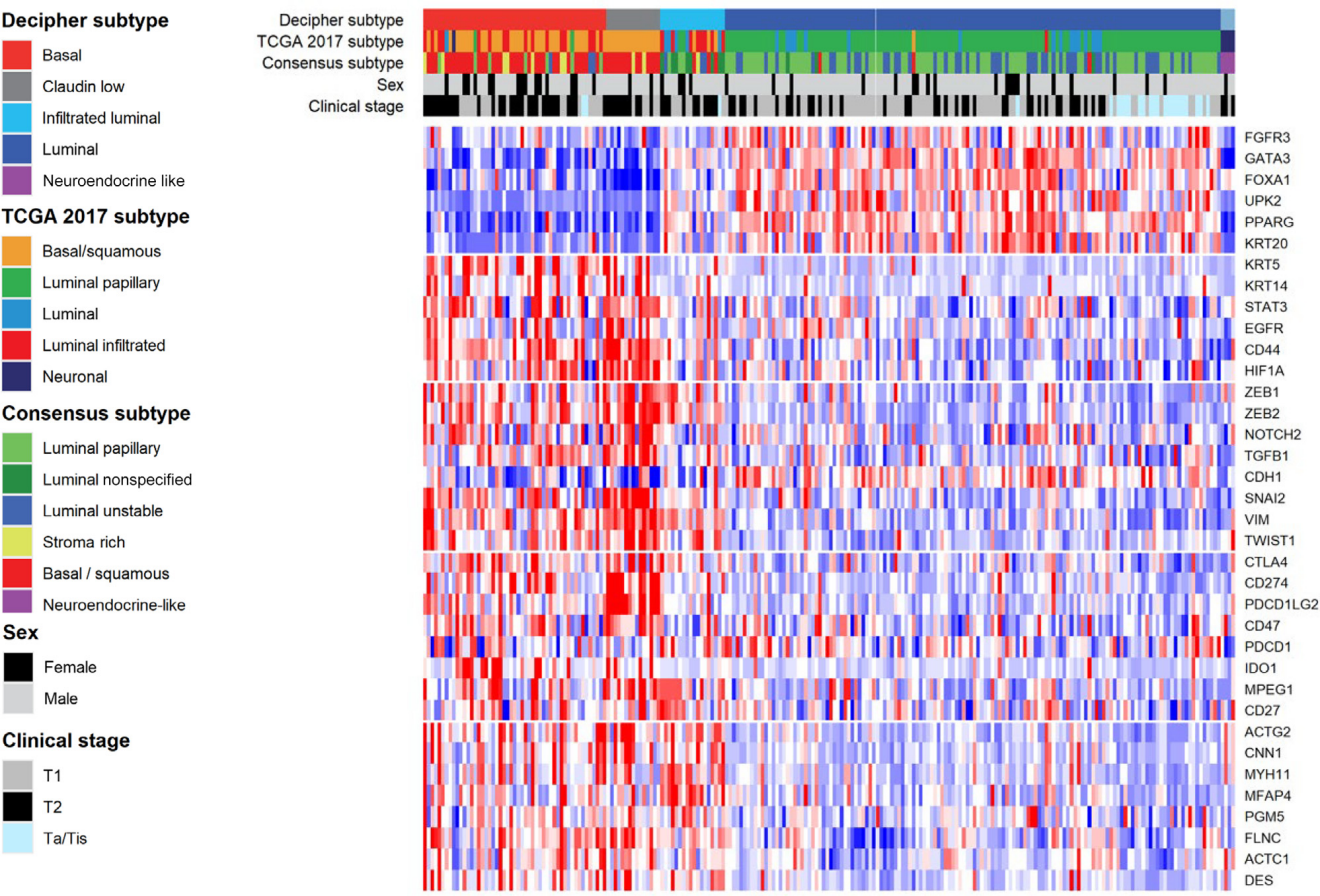
Forced order heatmap for five biological categories (luminal, basal, EMT, immune, and stromal) of selected bladder cancer marker genes. Female gender and clinical stage, with the TCGA [10], Consensus [10], and Decipher Bladder [7], [9] subtypes are indicated by black or colored bars in the respective covariate tracks. EMT = epithelial-mesenchymal transition; TCGA = The Cancer Genome Atlas.

Molecular subtyping revealed a GSC luminal subtype in 74% of patients with cNMIBC and in 42% of patients with cT2 (Supplementary Table 1). Comparing luminal versus nonluminal bladder cancer, luminal bladder cancer patients were younger at RC and had lower clinical stage (Table 1). Luminal tumors showed higher expression of markers typically associated with the luminal subtype (ie, *PPARG* and *KRT20*) and lower expression of basal (ie, *KRT5* and *KRT14*), epithelial-mesenchymal transition (ie, *ZEB1* and *VIM*), immune (ie, *CD274* and *CD8A*), and stromal (*MHY11* and *DES*) related genes (Fig. 1). Nonluminal tumors included 51 basal, 15 claudin-low, 18 infiltrated luminal, and 4 neuroendocrine-like tumors (Supplementary Table 2). Furthermore, TCGA and consensus subtype distributions were similarly associated with disease stage (Fig. 1 and Supplementary Table 2).

**Table 1:** Clinicopathological characteristics of the study cohort by GSC subtype

	Luminal (*n* = 138, 61%)	Nonluminal (*n* = 88, 39%)
Age at RC, median (IQR)	68 (60, 75)	73 (66, 78)
Sex, *n* (%)		
Female	20 (14)	22 (25)
Male	118 (86)	66 (75)
Smoking status, *n* (%)		
Never	30 (22)	21 (24)
Ever	92 (67)	55 (62)
Unknown	16 (12)	12 (14)
Clinical stage, *n* (%)		
Ta	8 (6)	0 (0)
Tis	13 (9)	3 (3)
T1	78 (57)	32 (36)
T2	39 (28)	53 (60)

GSC = genomic subtyping classifier; IQR = interquartile range; RC = radical cystectomy.

Some percentages do not add to 100% due to rounding.

### 3.3 Pathological upstaging to NOC disease at RC

Evaluating the primary endpoint, there were lower rates of upstaging to NOC disease in luminal tumors (38/138, 28%) than in nonluminal tumors (36/88, 41%; Table 2), which was significant on univariable but not on multivariable analysis (Table 3). Rates of upstaging to NOC disease were 16% versus 26% when comparing luminal versus nonluminal in the cNMIBC patient subgroup, and 56% versus 51% when comparing luminal versus nonluminal in the cT2 patient subgroup (Table 2). Supplementary Tables 3 and 4 list pathological tumor (pT) and nodal (pN) stage stratified by clinical stage, GSC subtype, and their combinations.

**Table 2:** Rates of pathological T stage upstaging, non–organ-confined disease, and node positivity by GSC subtype within clinical NMIBC and T2 patients

Subset	Variables	Luminal, *n* (%)	Nonluminal, *n* (%)
Overall	Total	138 (61)	88 (39)
	pT0–1	77 (56)	30 (34)
	pT2	33 (24)	26 (30)
	pT3–4	28 (20)	32 (36)
	pN+	21 (15)	17 (19)
	NOC (pT3–4 and/or pN+)	38 (28)	36 (41)
	MIBC+ (pT2+ and/or N+)	65 (47)	60 (68)
Clinical NMIBC	Total	99 (74)	35 (26)
	pT0–1	67 (88)	18 (80)
	pT2	20 (20)	10 (29)
	pT3–4	12 (12)	7 (20)
	pN+	8 (8)	4 (11)
	NOC (pT3–4 and/or pN+)	16 (16)	9 (26)
	MIBC+ (pT2+ and/or N+)	32 (32)	18 (51)
Clinical T2	Total	39 (42)	53 (58)
	pT0–1	10 (59)	12 (53)
	pT2	13 (33)	16 (30)
	pT3–4	16 (41)	25 (47)
	pN+	13 (33)	13 (25)
	NOC (pT3–4 and/or pN+)	22 (56)	27 (51)

GSC = genomic subtyping classifier; MIBC = muscle-invasive bladder cancer; NMIBC = non–muscle-invasive bladder cancer; NOC = non–organ confined.

**Table 3:** Univariable and multivariable logistic regression mixed model results for upstaging to NOC disease in the full cohort and upstaging to MIBC (pT2+ or N+) in the cNMIBC cohort, with site as a random effect

	Univariable		Multivariable	
	Odds ratio (95% CI)	*p* value	Odds ratio (95% CI)	*p* value
*Upstaging to NOC disease in the full cohort*				
GSC subtype: Nonluminal vs luminal	1.82 (1.03, 3.20)	0.04*	1.12 (0.59, 2.11)	0.74
Sex: male vs female	0.97 (0.47, 1.98)	0.93	1.04 (0.48, 2.24)	0.93
Age at RC (per 5 yr)	1.14 (0.98, 1.32)	0.08	1.07 (0.91, 1.25)	0.41
cT stage: cT2 vs NMIBC	4.97 (2.73, 9.03)	<0.001*	4.61 (2.45, 8.66)	<0.001*
*Upstaging to MIBC in the cNMIBC cohort*				
GSC subtype: nonluminal vs luminal	2.33 (1.04, 5.19)	0.04*	2.49 (1.09, 5.72)	0.03*
Sex: male vs female	1.91 (0.69, 5.25)	0.21	2.23 (0.78, 6.36)	0.13
Age at RC (per 5 yr)	1.07 (0.88, 1.29)	0.50	1.05 (0.86, 1.27)	0.64

CI = confidence interval; cNMIBC = clinically non–muscle-invasive bladder cancer; GSC = genomic subtyping classifier; MIBC = muscle-invasive bladder cancer; NMIBC = non–muscle-invasive bladder cancer; NOC = non–organ confined; RC = radical cystectomy.

### 3.4 Pathological upstaging to MIBC+ disease within clinical cNMIBC patients

Analyzing the secondary upstaging endpoint within the cNMIBC patient subgroup, fewer patients with luminal NMIBC were upstaged to MIBC+ disease (pT2+ and/or N+) at RC than those with nonluminal cNMIBC (32/99 [32%] vs 18/35 [51%]). On multivariable analyses, nonluminal tumors were significantly more likely to be upstaged to MIBC+ disease (adjusted odds ratio 2.49 [95% CI 1.09–5.72], *p* = 0.03; Table 3).

### 3.5 Nonluminal bladder cancer showed worse OS

**Fig. 2:**
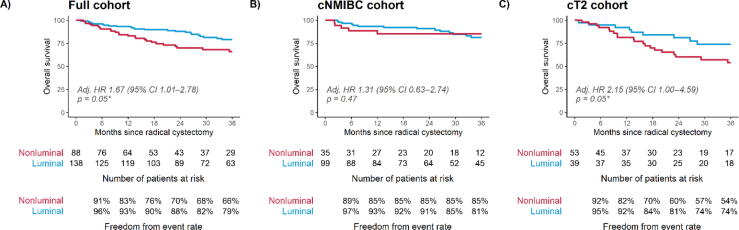
Kaplan-Meier plots for overall survival by GSC subtype in the (A) full cohort, (B) NMIBC cohort, and (C) cT2 cohort. Adjusted hazard ratios (Adj. HRs) and their corresponding 95% confidence intervals (CIs) are derived from multivariable models with sex, age, and stage (in the full cohort) included as covariates. GSC = genomic subtyping classifier; NMIBC = non–muscle-invasive bladder cancer.

Expectedly, patients who were upstaged to NOC disease had worse OS (Supplementary Fig. 1). Evaluating OS outcomes within the present cohort showed that patients with luminal subtype bladder cancer had better OS on multivariable analysis (nonluminal vs luminal adjusted HR 1.67 [95% CI 1.01–2.78], *p* = 0.05; Table 4 and Fig. 2A). Splitting the cohort further by clinical stage showed a larger survival difference within the cT2 bladder cancer subset (Fig. 2B and 2C), albeit with overlapping CIs.

**Table 4:** Univariable and multivariable Cox proportional hazard model results for overall survival in the full, cNMIBC, and cT2 cohorts

	Univariable		Multivariable	
	Hazard ratio (95% CI)	*p* value	Hazard ratio (95% CI)	*p* value
*Full cohort*				
GSC subtype: nonluminal vs luminal	1.79 (1.11–2.87)	0.02*	1.67 (1.01–2.78)	0.05*
Sex: male vs female	1.34 (0.66–2.71)	0.41	1.55 (0.76–3.16)	0.23
Age at RC (per 5 yr)	1.09 (0.96–1.24)	0.18	1.06 (0.93–1.21)	0.40
cT stage: cT2 vs NMIBC	1.61 (1.00–2.59)	0.05*	1.35 (0.82–2.23)	0.24
*cNMIBC cohort*				
GSC subtype: nonluminal vs luminal	1.24 (0.60–2.56)	0.56	1.31 (0.63–2.74)	0.47
Sex: male vs female	2.05 (0.62–6.75)	0.24	2.17 (0.66–7.22)	0.20
Age at RC (per 5 yr)	1.04 (0.87–1.24)	0.65	1.04 (0.86–1.25)	0.70
*cT2 cohort*				
GSC subtype: nonluminal vs luminal	2.07 (0.98–4.38)	0.06	2.15 (1.00–4.59)	0.05*
Sex: male vs female	1.11 (0.46–2.70)	0.81	1.31 (0.53–3.21)	0.56
Age at RC (per 5 yr)	1.08 (0.90–1.30)	0.42	1.08 (0.89–1.30)	0.45

CI = confidence interval; cNMIBC = clinically non–muscle-invasive bladder cancer; GSC = genomic subtyping classifier; NMIBC = non–muscle-invasive bladder cancer; RC = radical cystectomy.

## 4 Discussion

The aim of the present study was to validate that genomic subtyping can help predict upstaging in patients with clinically node-negative high-risk NMIBC and cT2 disease. We found that patients with luminal disease on TURBT specimens were less likely to have NOC disease at the time of RC. However, on a multivariate analysis that adjusted for clinical stage of disease, this association was not significant, largely because luminal patients were more likely to have NMIBC than nonluminal tumors. On the contrary, patients with nonluminal NMIBC were significantly more likely to be upstaged to muscle-invasive disease, and this study also reaffirmed the finding that patients with luminal subtype bladder cancer had better OS, which was statistically significant on the multivariable analysis.

There is a critical need to improve clinical staging for patients with bladder cancer. Prior studies found that over 40% of patients are understaged at the time of clinical staging, and approximately 25% are found to have occult nodal involvement at the time of cystectomy [3]. The present study confirmed a high rate of upstaging, with NOC disease being observed in 25 (19%) and 49 (53%) patients with cNMIBC and cT2 disease, respectively. Thirty-eight (17%) patients had positive lymph nodes at RC, including 12 (9%) and 26 (28%) patients with cNMIBC and cT2, respectively.

Much of the rationale for treating patients with systemic therapy is the concern for micrometastatic disease, and the greatest benefit for NAC has been found in patients with NOC disease [4]. The ability to improve the prediction regarding which patients have NOC could help select patients for these treatments and potentially enrich those who receive therapy with patients more likely to benefit. Studies from the National Cancer Database found that NAC use increased from 22.9% to 32.3% between 2011 and 2015, but these rates confirm that most patients with MIBC do not receive these therapies [11]. While this study did not find an independent association between subtyping and NOC (given the collinearity of luminal subtypes with clinical stage) on the multivariable analysis, we observed a significant association between subtyping and upstaging to NOC disease on the univariable analysis. In context, in our previous study of 206 patients with high-grade, cT1-T2, N0M0 UC, who underwent RC without NAC, upstaging to NOC was significantly lower for luminal than for nonluminal tumors, on the multivariable analysis [8].

Furthermore, the present study found that patients with luminal subtype bladder cancer had better OS, which was statistically significant on the multivariable analysis. This can have important implications regarding the need for adjuvant therapies and intensity of surveillance for these patients. Previous studies found similar favorable survival for luminal tumors. Seiler et al [7] developed a single-sample GSC using pre-NAC transurethral resection specimens from 343 patients with MIBC who underwent cystectomy and found that luminal tumors had the best OS with or without NAC. A more recent study of 601 patients with MIBC, including 247 who had been treated with NAC and cystectomy, and 354 who underwent cystectomy without NAC, evaluated the impact of a GSC on survival [6]. The study found an OS benefit with NAC versus cystectomy alone, but interestingly, the survival benefit was isolated to nonluminal tumor patients who had 10% greater OS at 3 yr, while luminal tumor patients had no difference in OS irrespective of whether they received NAC or had cystectomy alone. These studies raise several questions regarding whether a patient with luminal subtype should receive NAC based on both a lower likelihood of NOC disease and potentially less benefit for biological reasons.

Similarly, it suggests that patients with nonluminal disease should be encouraged to undergo NAC since they may be more likely to benefit. Prospective randomized trials to evaluate this will be necessary to provide definitive evidence, but there is a growing body of evidence that may support using these markers in discussion with patients regarding the pros and cons of pursuing more aggressive therapies. Of note, other such markers are currently used in prostate cancer without the support of randomized trials. Interestingly, GUSTO presently evaluates omitting NAC in TCGA luminal-papillary MIBC patients [12], a tumor subtype concordant with the GSC luminal subtype [5].

There are limitations that are inherent in retrospective studies. Many of the centers are referral centers, and TURBT was performed outside the institutions for some of the patients. Most patients underwent restaging prior to cystectomy, but this was not uniformly captured in the clinical databases. Furthermore, the data lacked information on intravesical/adjuvant therapies, and there is a selection bias regarding which patients should undergo cystectomy for high-risk NMIBC, or cystectomy without neoadjuvant therapy; however, absence of NAC was a necessary inclusion criterion since NAC would impact the ability to evaluate the rates of upstaging. While some analyses were performed separately in patients with NMIBC and cT2 disease, we were underpowered to perform any formal interaction analyses.

## 5 Conclusions

Molecular subtyping in cNMIBC suggests that luminal tumors harbor less aggressive disease, as reflected by lower rates of pathological upstaging to MIBC and/or pN+. In addition, in MIBC, luminal tumors were associated with favorable outcomes with RC, even in the absence of systemic NAC, validating previous findings.

  ***Author contributions:*** Yair Lotan had full access to all the data in the study and takes responsibility for the integrity of the data and the accuracy of the data analysis.

  *Study concept and design*: Gibb, Lotan.

*Acquisition of data*: Daneshmand, Svatek, Naryan, Joshi, Dahmen, Li, Inman, Shah, Chaplin, Wright, Lotan.

*Analysis and interpretation of data*: de Jong, Proudfoot, Davicioni, Lotan.

*Drafting of the manuscript*: de Jong, Proudfoot, Davicioni, Lotan.

*Critical revision of the manuscript for important intellectual content*: All authors.

*Statistical analysis*: Proudfoot.

*Obtaining funding*: Lotan, Davicioni.

*Administrative, technical, or material support*: Davicioni, Gibb.

*Supervision*: Lotan.

*Other*: None.

  ***Financial disclosures:*** Yair Lotan certifies that all conflicts of interest, including specific financial interests and relationships and affiliations relevant to the subject matter or materials discussed in the manuscript (eg, employment/affiliation, grants or funding, consultancies, honoraria, stock ownership or options, expert testimony, royalties, or patents filed, received, or pending), are the following: Dr. Yair Lotan is a consultant for Nanorobotics, Photocure, AstraZeneca, Merck, Fergene, Abbvie, Nucleix, Ambu, Seattle Genetics, Hitachi, Ferring Research, Verity Pharmaceutics, Virtuoso Surgical, Stimit, Urogen, Vessi Medical, CAPs Medical, Xcures, BMS, Nonagen, Aura Biosciences, Inc., Convergent Genomics, Pacific Edge, Pfizer, Phinomics Inc, CG Oncology, Uroviu, On Target Lab, Promis Diagnostics, Valar Labs, Uroessentials, NRx Pharmaceuticals, Vesica Health, Janssen, and Immunity Bio. Joep J. de Jong is a consultant for Veracyte Inc. Dr. Siamak Daneshmand is a consultant for Photocure, Pacific Edge, Ferring, Johnson & Johnson, Protara, Urogen, Pfizer, CG Oncology, Vesica Health, and Immunitybio. Brant Inman: clinical trials and research agreements with Genentech/Roche, FKD Therapies, Taris Biomedical, Seattle Genetics, Medtronic, Janssen, CG Oncology, Profound Medical, and Theralase; advisor for Combat Medical, Johnson & Johnson, TerSera, and AbbVie. Dr. Robert S. Svatek is a consultant for CG Oncology and receives research support from Merck, Japanese BCG Laboratories, Biodexa/Emtora, Valar Labs, and Veracyte. Roger Li: research support—Predicine, Veracyte, CG Oncology, Valar Labs, and Merck; clinical trial protocol committee—CG Oncology, Merck, and Janssen; and scientific advisor/consultant—BMS, Merck, Fergene, Arquer Diagnostics, Urogen Pharma, Lucence, CG Oncology, Janssen, and Thericon. James A. Proudfoot and Elai Davicioni are employees of Veracyte Inc. Ewan A. Gibb is a former employee of Veracyte Inc.

  ***Funding/Support and role of the sponsor:*** Veracyte Inc. provided Decipher Bladder transcriptome-wide analyses for the study. Yair Lotan acknowledges the Wilson Foundation for financial support for study conduct. Joep J. de Jong acknowledges the Erasmus MC Young Investigator Grant.
