# Supplementary material for: Molecular Subtyping for Predicting Pathological Upstaging and Survival Outcomes in Clinically Organ-confined Bladder Cancer Patients Undergoing Radical Cystectomy
Source: Eur Urol Open Sci. 2025 Jan 27;73:24–30. doi: 10.1016/j.euros.2024.12.009 (PMC11810690; doi:10.1016/j.euros.2024.12.009)

**Supplementary Table 1:** Clinicopathologic characteristics of the study cohort by clinical staging.

|  | **cNMIBC** (n = 134, 59%) | **cT2** (n = 92, 41%) |
| --- | --- | --- |
| **Age at RC; median (IQR)** | 69 (60, 75) | 73 (65, 79) |
| **Sex; n (%)** |  |  |
| Female | 24 (18%) | 18 (20%) |
| Male | 110 (82%) | 74 (80%) |
| **Smoking status; n (%)** |  |  |
| Never | 34 (25%) | 17 (18%) |
| Ever | 80 (60%) | 67 (73%) |
| Unknown | 20 (15%) | 8 (9%) |
| **GSC subtype; n (%)** |  |  |
| Luminal | 99 (74%) | 39 (42%) |
| Non-luminal | 35 (26%) | 53 (58%) |

**Supplementary Table 2:** Subtype distributions for different subtyping classifiers by clinical stage.

|  | **cNMIBC** (n = 134, 59%) | **cT2** (n = 92, 41%) |
| --- | --- | --- |
| **GSC subtype** |  |  |
| Luminal | 99 (74%) | 39 (42%) |
| Non-luminal | 35 (26%) | 53 (58%) |
| **TCGA subtype** |  |  |
| Luminal Papillary | 94 (70%) | 30 (33%) |
| Luminal | 9 (7%) | 13 (14%) |
| Luminal Infiltrated | 12 (9%) | 13 (14%) |
| Basal Squamous | 18 (13%) | 32 (35%) |
| Neuronal | 1 (1%) | 4 (4%) |
| **Consensus subtype** |  |  |
| Luminal P | 70 (52%) | 22 (24%) |
| Luminal NS | 7 (5%) | 4 (4%) |
| Luminal U | 34 (25%) | 22 (24%) |
| Basal Squamous | 17 (13%) | 36 (39%) |
| Stroma Rich | 5 (4%) | 5 (5%) |
| NE-Like | 1 (1%) | 3 (3%) |

**Supplementary Table 3:** Pathological stage by clinical stage, GSC subtype, and their combination

|  | **Pathological stage** | | | | | |
| --- | --- | --- | --- | --- | --- | --- |
|  | pT0 | pTa/pTis | pT1 | pT2 | pT3 | pT4 |
| Total | 11 (5%) | 37 (16%) | 59 (26%) | 59 (26%) | 47 (21%) | 13 (6%) |
| **Clinical stage and GSC; n (%)** |  |  |  |  |  |  |
| NMIBC Luminal | 5 (45%) | 23 (62%) | 39 (66%) | 20 (34%) | 9 (19%) | 3 (23%) |
| NMIBC Non-luminal | 2 (18%) | 7 (19%) | 9 (15%) | 10 (17%) | 3 (6%) | 4 (31%) |
| cT2 Luminal | 1 (9%) | 2 (5%) | 7 (12%) | 13 (22%) | 15 (32%) | 1 (8%) |
| cT2 Non-luminal | 3 (27%) | 5 (14%) | 4 (7%) | 16 (27%) | 20 (43%) | 5 (38%) |
| **Clinical stage (granular); n (%)** |  |  |  |  |  |  |
| Ta | 0 (0%) | 7 (19%) | 1 (2%) | 0 (0%) | 0 (0%) | 0 (0%) |
| Tis | 0 (0%) | 0 (0%) | 7 (12%) | 7 (12%) | 1 (2%) | 1 (8%) |
| T1 | 7 (64%) | 23 (62%) | 40 (68%) | 23 (39%) | 11 (23%) | 6 (46%) |
| T2 | 4 (36%) | 7 (19%) | 11 (19%) | 29 (49%) | 35 (74%) | 6 (46%) |
| **Clinical stage; n (%)** |  |  |  |  |  |  |
| cNMIBC | 7 (64%) | 30 (81%) | 48 (81%) | 30 (51%) | 12 (26%) | 7 (54%) |
| cT2 | 4 (36%) | 7 (19%) | 11 (19%) | 29 (49%) | 35 (74%) | 6 (46%) |
| **GSC subtype; n (%)** |  |  |  |  |  |  |
| Luminal | 6 (55%) | 25 (68%) | 46 (78%) | 33 (56%) | 24 (51%) | 4 (31%) |
| Non-luminal | 5 (45%) | 12 (32%) | 13 (22%) | 26 (44%) | 23 (49%) | 9 (69%) |
| **GSC subtype (granular); n (%)** |  |  |  |  |  |  |
| Luminal | 6 (55%) | 25 (68%) | 46 (78%) | 33 (56%) | 24 (51%) | 4 (31%) |
| Infiltrated luminal | 0 (0%) | 7 (19%) | 4 (7%) | 5 (8%) | 1 (2%) | 1 (8%) |
| Basal | 4 (36%) | 3 (8%) | 8 (14%) | 15 (25%) | 15 (32%) | 6 (46%) |
| Claudin low | 1 (9%) | 1 (3%) | 1 (2%) | 5 (8%) | 6 (13%) | 1 (8%) |
| NE-like | 0 (0%) | 1 (3%) | 0 (0%) | 1 (2%) | 1 (2%) | 1 (8%) |

**Supplementary Table 4:** Pathological node status by clinical stage, GSC subtype, and their combination.

|  | **Pathological node status** | | | | |
| --- | --- | --- | --- | --- | --- |
|  | N0 | N1 | N2 | N3 | Unknown |
| Total | 187 (83%) | 21 (9%) | 16 (7%) | 1 (0%) | 1 (0%) |
| **Clinical stage and GSC; n (%)** |  |  |  |  |  |
| NMIBC Luminal | 90 (48%) | 5 (24%) | 3 (19%) | 0 (0%) | 1 (100%) |
| NMIBC Non-luminal | 31 (17%) | 3 (14%) | 1 (6%) | 0 (0%) | 0 (0%) |
| cT2 Luminal | 26 (14%) | 6 (29%) | 6 (38%) | 1 (100%) | 0 (0%) |
| cT2 Non-luminal | 40 (21%) | 7 (33%) | 6 (38%) | 0 (0%) | 0 (0%) |
| **Clinical stage (granular); n (%)** |  |  |  |  |  |
| Ta | 8 (4%) | 0 (0%) | 0 (0%) | 0 (0%) | 0 (0%) |
| Tis | 14 (7%) | 2 (10%) | 0 (0%) | 0 (0%) | 0 (0%) |
| T1 | 99 (53%) | 6 (29%) | 4 (25%) | 0 (0%) | 1 (100%) |
| T2 | 66 (35%) | 13 (62%) | 12 (75%) | 1 (100%) | 0 (0%) |
| **Clinical stage; n (%)** |  |  |  |  |  |
| cNMIBC | 121 (65%) | 8 (38%) | 4 (25%) | 0 (0%) | 1 (100%) |
| cT2 | 66 (35%) | 13 (62%) | 12 (75%) | 1 (100%) | 0 (0%) |
| **GSC subtype; n (%)** |  |  |  |  |  |
| Luminal | 116 (62%) | 11 (52%) | 9 (56%) | 1 (100%) | 1 (100%) |
| Non-luminal | 71 (38%) | 10 (48%) | 7 (44%) | 0 (0%) | 0 (0%) |
| **GSC subtype (granular); n (%)** |  |  |  |  |  |
| Luminal | 116 (62%) | 11 (52%) | 9 (56%) | 1 (100%) | 1 (100%) |
| Infiltrated luminal | 17 (9%) | 0 (0%) | 1 (6%) | 0 (0%) | 0 (0%) |
| Basal | 40 (21%) | 6 (29%) | 5 (31%) | 0 (0%) | 0 (0%) |
| Claudin low | 11 (6%) | 3 (14%) | 1 (6%) | 0 (0%) | 0 (0%) |
| NE-like | 3 (2%) | 1 (5%) | 0 (0%) | 0 (0%) | 0 (0%) |

**Supplementary Figure 1:** Kaplan-Meier plot of overall survival by NOC disease at RC.


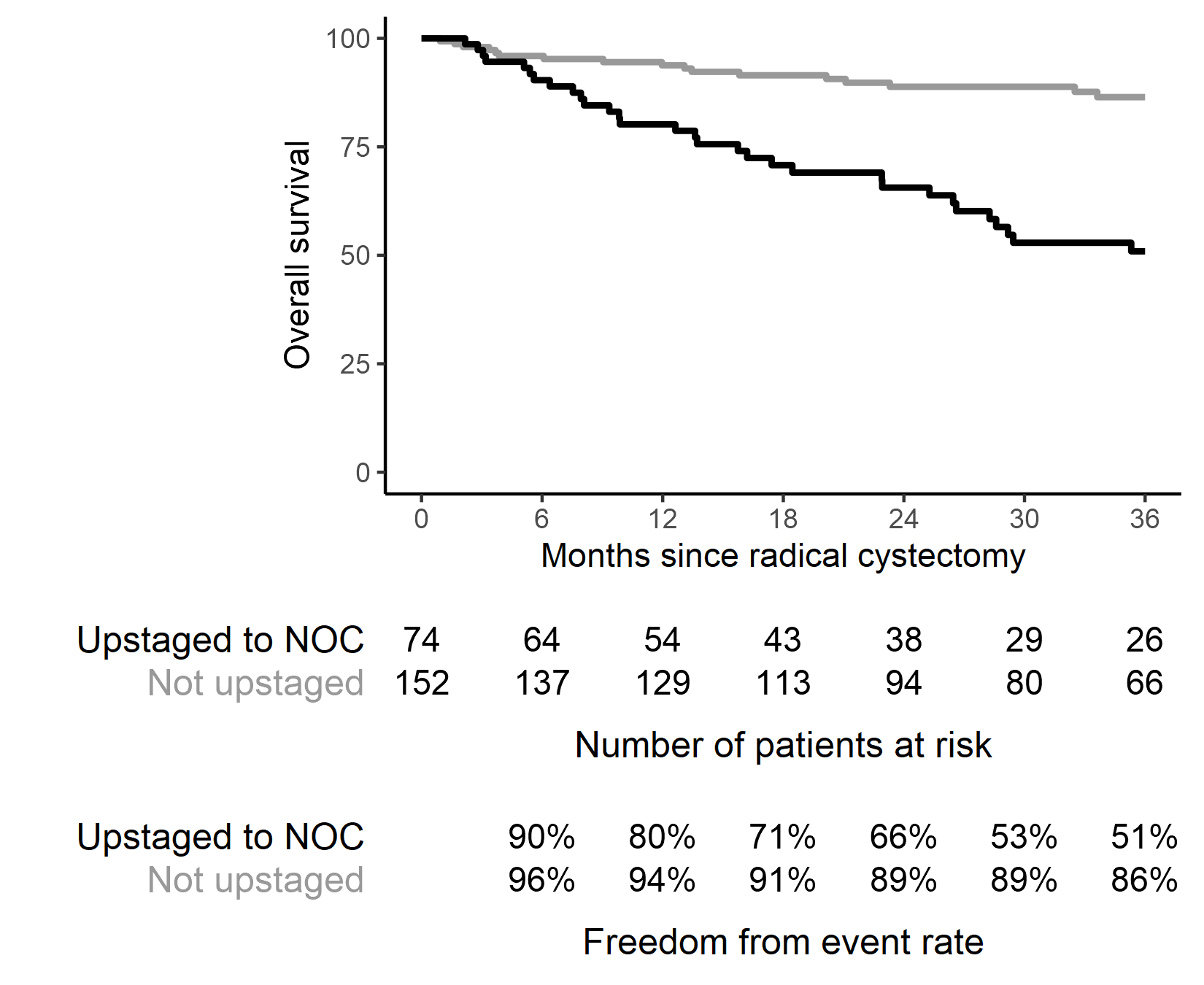

Supplement: Supplementary Data 1 [file mmc1.docx]
